# THE CELL “SURFACEOME”: A STRATEGY TO TARGET SENESCENT CELLS IN VIVO

**DOI:** 10.1093/geroni/igad104.1939

**Published:** 2023-12-21

**Authors:** Reema Banarjee, Amit Dey, Thedoe Nyunt, Jordan Gregg, Dimitrios Tsitsipatis, Allison Herman, Myriam Gorospe, Nathan Basisty

**Affiliations:** National Institute on Aging, National Institutes of Health, Baltimore, Maryland, United States; National Institute on Aging, National Institutes of Health, Baltimore, Maryland, United States; National Institute on Aging, National Institutes of Health, Baltimore, Maryland, United States; National Institute on Aging, National Institutes of Health, Baltimore, Maryland, United States; National Institute on Aging, National Institutes of Health, Baltimore, Maryland, United States; National Institute on Aging, National Institutes of Health, Baltimore, Maryland, United States; National Institute on Aging, National Institutes of Health, Baltimore, Maryland, United States; National Institute on Aging, National Institutes of Health, Baltimore, Maryland, United States

## Abstract

Senescent cell accumulation drives aging and related pathologies including metabolic disorders and neurodegeneration, and their selective elimination is a promising therapeutic approach to treat multiple diseases of aging in humans. However, developing drugs to eliminate senescent cells requires specific biomarkers to selectively identify and target these cells in vivo. In this study, we have performed a comprehensive and quantitative proteomic profiling of surface proteomes (surfaceomes) of senescent cells to identify potential senescence biomarker candidates in human tissues. We induced senescence using ionizing radiation in a variety of cell types including lung fibroblasts, monocytes, vascular smooth muscle cells, aortic endothelial cells and preadipocytes. Senescence was validated using a panel of canonical markers including reduced proliferation, increased senescence-associated β-galactosidase activity, and increased expression of p21, p16, IL6, and others. The cell surface proteome was enriched using an optimized surface biotinylation approach (Glyco-cell surface capture) followed by data-independent acquisition (DIA) LC-MS/MS analysis on a Q-Exactive HF Orbitrap mass spectrometer. Our analysis revealed novel cell surface targets that were further validated by flow cytometry and immunohistochemistry. These results provide clinically useful information for establishing biomarkers and therapeutic targets to aid in the translation of senescence-targeted therapies to treat age-associated diseases.

